# The value of next-generation metagenomic sequencing in pathogen detection of pleural effusions and ascites from children with sepsis

**DOI:** 10.3389/fcimb.2023.1130483

**Published:** 2023-02-20

**Authors:** Gang Liu, Lijuan Wang, Xuming Li, Ye Zhang, Hu Long, Yi Wang, Hengmiao Gao, Han Xia, Suyun Qian

**Affiliations:** ^1^ Department of Pediatric Intensive Care Unit, Beijing Children’s Hospital, Capital Medical University, National Center for Children’s Health, Beijing, China; ^2^ Department of scientific affairs, HugoBiotech Co., Ltd., Beijing, China

**Keywords:** metagenomic next-generation sequencing (mNGS), pathogen detection, children, pleural effusions and ascites, sepsis

## Abstract

**Objective:**

To investigate the diagnostic value of metagenomic next-generation sequencing (mNGS) using pleural effusion and ascites from children with sepsis.

**Methods:**

In this study, children with sepsis or severe sepsis and appeared pleural or peritoneal effusions were enrolled, of whom the pleural effusions or ascites and blood samples were conducted pathogen detection using both conventional and mNGS methods. The samples were divided into pathogen-consistent and pathogen-inconsistent groups based on the consistency of mNGS results from different sample types, and into exudate and transudate groups based on their pleural effusion and ascites properties. The pathogen positive rates, pathogen spectrum, consistency between different sample types, and clinical diagnosis consistency were compared between mNGS and conventional pathogen tests.

**Results:**

A total of 42 pleural effusions or ascites and 50 other type samples were collected from 32 children. The pathogen positive rate of the mNGS test was significantly higher than that of traditional methods (78.57% *vs*. 14.29%, *P* < 0.001**)** in pleural effusion and ascites samples, with a consistent rate of 66.67% between the two methods. Nearly 78.79% (26/33) of mNGS positive results of the pleural effusions and ascites samples were consistent with clinical evaluation, and 81.82% (27/33) of these positive samples reported 1-3 pathogens. The pathogen-consistent group outperformed the pathogen-inconsistent group in terms of consistency with respect to clinical evaluation (88.46% *vs*. 57.14%, *P* = 0.093), while there was no significant difference between the exudate and transudate groups (66.67% *vs*. 50.00%, *P* = 0.483).

**Conclusion:**

Compared to conventional methods, mNGS has great advantages in pathogen detection of pleural effusion and ascites samples. Moreover, consistent results of mNGS tests with different sample types provide more reference values in clinical diagnosis.

## Background

Sepsis has a high morbidity and mortality in the pediatric population, and effective anti-infective treatment is crucial to increase the survival rate (Fleischmann-Struzek et al., 2018). However, traditional techniques for pathogen detection are often time-consuming and have low positive rates, leading to additional reliance on experience in identifying infections and targeted anti-infectious therapy. Metagenomics next generation sequencing (mNGS) test is rapid, highly sensitive, and less affected by previous treatment. Although there are still some drawbacks, it has been shown to have significant advantages over conventional methods in the detection of body fluids such as blood, sputum, and cerebrospinal fluid (CSF) (Miller et al., 2019; Zinter et al., 2019).

Sepsis tends to develop more quickly in children than in adults. Therefore, conducting rapid and comprehensive pathogen screening of biological samples from a variety of suspected infection foci, such as cerebral fluid, pleural effusion, or ascites, is a highly important method to improve the accuracy of pathogen diagnosis and reduce the fatality rate. Currently, mNGS pathogen detection technique offers the possibility to realize this clinical demand. Children with sepsis in pediatric intensive care unit (PICU) are mostly complicated with underlying diseases and multiple organ dysfunction. Pleural or peritoneal effusions are common symptom of infectious diseases in children (Karnsakul et al., 2017) and can lead to a poor prognosis and even death (Piotrowski et al., 2006; Khan et al., 2009; Kookoolis et al., 2014). The formation mechanism of pleural and peritoneal effusion is complex, and whether it is secondary to infection is difficult to determine, and the detection of causative agents remains the gold standard. Traditional pathogen detection methods are far from meeting clinical needs due to their limitations (Huang et al., 2020). Though mNGS is an increasingly emerging and relatively ideal auxiliary detection method, the value of mNGS for pathogen detection in pleural and peritoneal effusions in children with sepsis are not completely understood due to the small number of relevant studies.

In this study, we collected pleural and peritoneal effusion samples as well as other sample types simultaneously from 32 children with sepsis and hospitalized in PICU.

For the first time, this study described the positive rate of mNGS pathogen detection in pleural effusions and ascites samples of children with sepsis, and pathogen consistency and clinical consistency were also compared with those of other samples, aiming at providing valuable evidence for the values of clinical mNGS pathogen diagnosis in pleural and abdominal fluid samples.

## Materials and methods

### Samples and data collection

Children with sepsis or severe sepsis who underwent mNGS test of pleural or peritoneal effusions in the PICU of Beijing Children’s Hospital were enrolled. The inclusion criteria: 1) met the diagnostic criteria for sepsis and severe sepsis as defined by the 2005 International Sepsis Guidelines for Children (Goldstein et al., 2005); 2) aged between 29 days and 18 years old; 3) hospitalized at the PICU of Beijing Children’s Hospital; 4) had pleural or peritoneal effusion samples collected for pathogen detection by mNGS; 5) underwent etiological examination by other methods such as culture of samples from the same site at the same time, or underwent mNGS test of the body fluid samples collected from different sites at the same time. The exclusion criteria: 1) had only pleural or peritoneal effusions but no additional site specimens sent for examination in the same pathogen screening by mNGS detection; 2) had only mNGS test but no traditional test results for the same biological sample; 3) had only electronic case records of mNGS results but no original reports; 4) had incomplete clinical data. This study was a secondary analysis based on the data of a prospective study of severe sepsis in children (2021.11∼2023.12) (Beijing Children’s Hospital Ethical Approval No. [2022] -E-019-Y). mNGS results of pleural or peritoneal effusion samples from a total of 45 pediatric patients were collected, from which 32 children who met the inclusion and exclusion criteria were selected. Clinical data, including demographic information, organ dysfunction, underlying disease, laboratory tests, results of conventional and mNGS tests, and in-hospital mortality, were collected in this study.

### Grouping method

Patients were divided into exudate and transudate groups based on the original state of their pleural and peritoneal effusions. Pleural effusion was judged to be exudate if it met at least one item of the Light criteria, otherwise it was judged to be transudate. The Light criteria (Light et al., 1972) are total protein in pleural fluid/total protein in serum > 0.5, LDH in pleural fluid/LDH in serum > 0.6 and LDH activity in pleural fluid ≥ 200 U/L. Peritoneal effusion was judged as exudate if it met at least two items of Boyer’s criteria, otherwise it was judged as transudate. The Boyer’s criteria (Boyer et al., 1978) are total protein in ascitic fluid/total protein in serum ≥ 0.5, LDH in ascitic fluid/LDH in serum ≥ 0.6 and LDH activity in pleural fluid ≥ 400 U/L.

Clinical coincidence: if at least one pathogen in the mNGS results of pleural or peritoneal effusion samples conformed to the clinical judgment, the current diagnosis and treatment plan were adjusted or confirmed according to the positive or negative results, it was judged as a clinical coincidence, and vice versa.

If a same microorganism is positive in pleural effusions or ascites and other sample types collected at the same time, it’s more likely to be the pathogenic pathogen, which can improve the pathogen diagnosis accuracy. In this study, pathogen-consistent and pathogen-inconsistent groups were set to further clarify whether the detection of the same pathogen can benefit the children more. If at least one pathogen in the mNGS results of pleural or peritoneal effusions was consistent with the pathogen detected in other samples, it was judged to be pathogen-consistent, and vice versa.

### mNGS pathogen detection

The clinical samples were performed PACEseq mNGS test by Hugobiotech Co., Ltd. During the experimental process, negative controls (sterile deionized water) and positive controls (synthesize fragments with known quantities) were established for each batch of experiments using the same wet lab procedures and bioinformatics analysis as the clinical samples. The read number and reads per million (RPM) of each detected microbe were calculated. For detected microbes, including bacteria (*Mycobacterium* excluded), fungi (*Cryptococcus* excluded), and parasites, a positive mNGS result was given when its coverage ranked in the top 10 of similar microbial species (or genera) and was absent in the negative control (“No template” control, NTC) or when its ratio of RPM between sample and NTC (RPMsample/RPMNTC) > 10 if RPMNTC≠0. For viruses, *Mycobacterium*, and *Cryptococcus*, a positive mNGS result was considered when at least one unique read was mapped to species level and absent in NTC or RPM sample/RPMNTC > 5 when RPMNTC≠0.

### Statistical analysis

Statistical analysis was performed using SPSS 24.0 statistical software. Measurement data in accordance with normal distribution were expressed as X ± s, and one-way analysis of variance was used for comparison between groups. Measurement data in the non-normal distributions were expressed as median (interquartile range) [M (P25, P75)], and the Mann-Whitney U test was used for comparison between groups. Enumeration data were expressed as percentages (%), and χ2 test or Fisher exact test was used for comparison between groups. *P*<0.05 was considered statistically significant.

## Results

### Clinical features and mNGS samples

**Figure 1:**
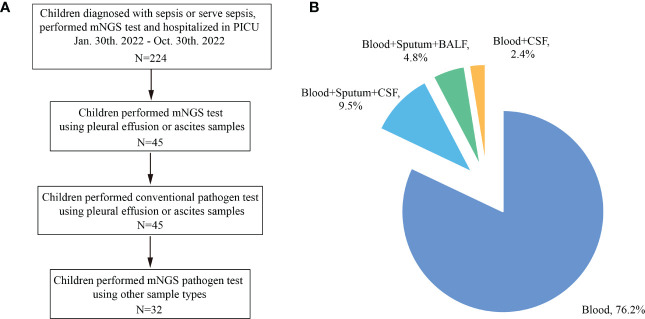
Sampling strategy. **(A)** flow graph showing the patient screening procedures; **(B)** pie graph showing the performed mNGS test of included patients.

A total of 32 pediatric patients with sepsis were included in the study (**Figure 1A**). The average age was 7 (3, 10) years old, and 59.38% (19/32) were male. Their major organ dysfunctions were respiratory insufficiency (53.13%), acute kidney injury (46.88%), and septic shock (34.38%). The medians of procalcitonin (PCT) and C-reaction protein (CRP) levels were 16.0 (1.5, 58.5) ug/L and 14.0 (1.8, 80.5) mg/L, respectively. 81.25% (26/32) of the patients had underlying diseases, with tumors accounting for 50.00% (13/26). In addition, the in-hospital mortality was 18.75% (6/32) (**Tables 1**, **S1**).

**Table 1:** Clinical features and laboratory tests of involved patients (N=32).

Clinical features	Values
Age [years, M (Q1, Q3)]	7 (3, 10)
Gender [male, %]	19 (59.4)
Organ dysfunction [case, %]	
Respiratory insufficiency/failure	17 (53.13)
Acute kidney injury	15 (46.88)
Septic shock	11 (34.38)
Cardiac insufficiency/failure	9 (28.13)
Brain dysfunction	7 (21.88)
Gastrointestinal failure	2 (6.25)
Hepatic failure	1 (3.12)
Disseminated coagulation disorders	1 (3.12)
Laboratory tests [M (Q1, Q3)]	
PCT [μg/L, M (Q1, Q3)]	16.0 (1.5, 58.5)
WBC [×10^9^/L, M (Q1, Q3)]	6.4 (4.0,12.9)
CRP [mg/L, M (Q1, Q3)]	14.0 (1.8, 80.5)
Underlying disease [case, %]	26 (81.25)
Tumor	13 (40.63)
Malnutrition	8 (25.00)
Low immunity	6 (18.75)
Others ^a^	4 (12.5)
In-hospital death [case, %]	6 (18.75)

a Others included one case each of lysosomiasis, dermatomyositis, Langerhans cell histiocytosis, and congenital neutropenia.

A total of 42 pleural effusions or ascites samples were obtained in this study. Four patients simultaneously collected pleural effusions and ascites samples. Four patients had two, and one patient had three ascites samples collected. Any pleural effusion or ascites sample was regarded as an independent test. Among them, 76.19% (32/42) were detected only in combination with blood samples, 9.52% (4/42) were detected in combination with blood, deep sputum, and CSF, 4.76% (2/42) were detected in combination with blood, deep sputum, and bronchoalveolar lavage fluid (BALF), and 2.38% (1/42) were detected in combination with blood and CSF (**Figure 1B**; **Table S2**).

### Positive rates of conventional and mNGS tests

**Figure 2:**
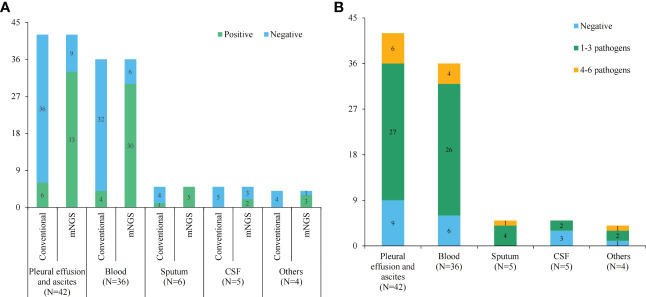
The statistics of conventional and mNGS pathogen tests. **(A)** The positive and negative rates of conventional and mNGS pathogen tests in different sample types; **(B)** histogram showing detected pathogens of mNGS test in different sample types.

The positive rates of pathogens in pleural or peritoneal effusions, blood, sputum, CSF and other body fluids by the conventional method were 14.29% (6/42), 11.11% (4/36), 20.00% (1/5), 0 and 0, respectively. However, the positive rates by mNGS were 78.57% (33/42), 83.33% (30/36), 100.00% (5/5), 40.00% (2/5) and 75.00% (3/4), respectively, which were great higher than those of the conventional test (*P* < 0.05) (**Figure 2A**). Furthermore, mNGS detected more pathogens at a single test in comparison to the conventional methods. Only 3.26% (3/92) of the conventional tests returned results for multi-pathogens. The proportions of 1-3 pathogens detected in pleural and peritoneal effusion, blood, deep sputum, and other body fluid were 64.29% (27/42), 72.22% (26/36), 80.00% (4/5), 40.00% (2/5) and 50.00% (2/4), respectively, while the proportions of 4-6 pathogens detected were 14.28 (6/42), 11.11% (4/36), 20.00% (1/5), 0 and 25.00% (1/4) (**Figure 2B**).

### Consistency between conventional and mNGS tests

**Figure 3:**
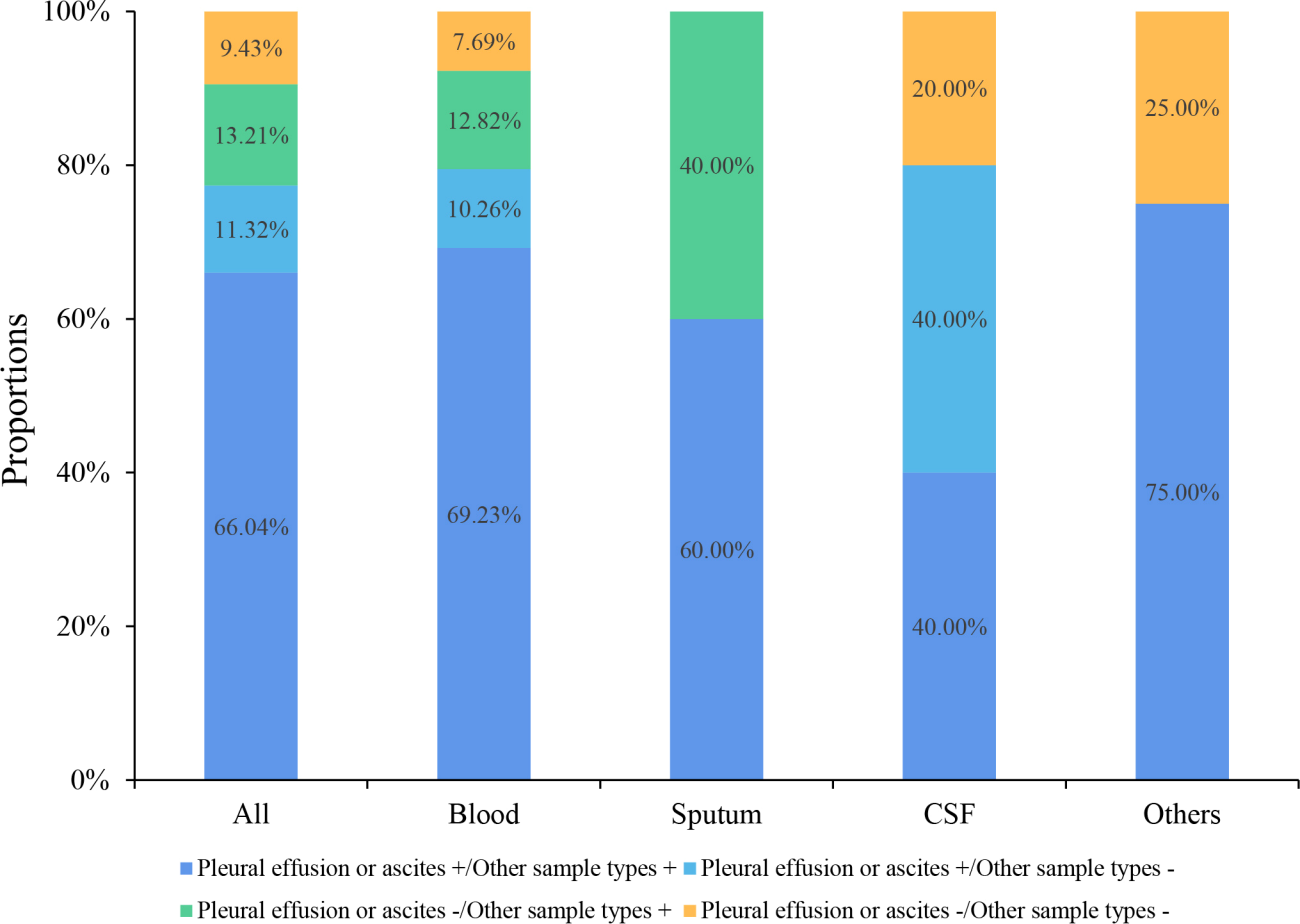
Consistency of mNGS test results between pleural effusions or ascites and the other sample types.

**Figure 4:**
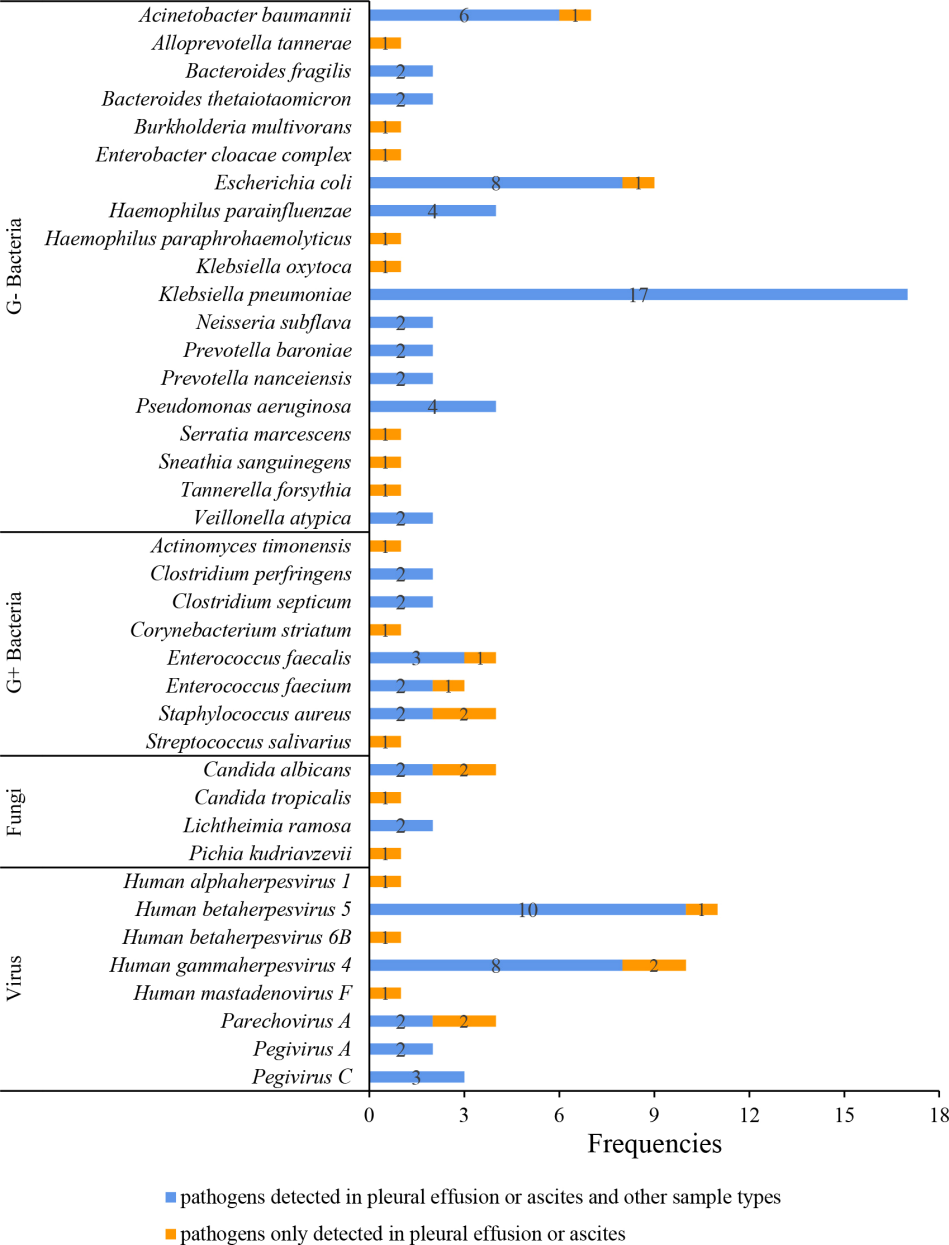
Pathogen spectrum detected by mNGS in different sample types.

Six (14.29%, 6/42) pleural effusion or ascites samples were tested positive by traditional culture, 66.67% (4/6) of which were consistent with mNGS results. Four blood and one deep sputum samples were tested positive by traditional culture, and the consistency rate with mNGS results was 100% (4/4, 1/1). The consistencies of mNGS results between pleural effusions or ascites and other samples are shown in **Figure 3**. The proportion of the same pathogens detected in pleural effusions or ascites as in blood, deep sputum, bronchoalveolar lavage fluid, and other body fluid samples were 69.23% (27/39), 60.00% (3/5), 40.00% (2/5), and 75.00% (3/4), respectively. A total of 11 G- bacteria, 5 G+ bacteria, 2 fungi and 5 viruses were simultaneously detected in blood and other sample types. It’s worth noting that 8 G- bacteria, 3 G+ strains, 2 fungi and 3 viruses were only detected in pleural or peritoneal effusions (**Figure 4**).

### Clinical consistency between the pathogen-consistent and pathogen-inconsistent groups

Most of the patients were collected with one set of samples. Four patients with two sets of samples and one patient with three sets of samples were grouped into pathogen-consistent group. A patient with two sets of data from were separated into two groups. The PCT (16 *vs*. 2.3, *P* = 0.141) and CRP (14 *vs*. 11, *P* = 0.401) levels were both higher in the pathogen-consistent group than in the pathogen-inconsistent group but showed no significance. The pathogens detected in 27 pleural effusions or ascites samples were consistent with clinical findings, suggesting that infectious pleural or peritoneal effusion samples accounted for 64.29% (27/42). Among them, 85.2% (23/27) of the detected pathogens in the pathogen-consistent group were consistent with the clinical judgment, which was higher than it (57.0%) in the pathogen-inconsistent group (*P* = 0.093). There was no significant difference in mortality and adjustment rate of antibiotic therapies between the two groups (**Table 2**).

**Table 2:** Comparison of clinical features and pathogen constitution between the pathogen-consistent group and pathogen-inconsistent group.

Test results	Pathogen inconsistent group (N=6)	Pathogen consistent group (N=27)	X^2^/Z	P value
PCT [μg/L, M (Q1, Q3)]	2.3 (1.1, 29)	16 (4.3, 79)	1.472	0.141
CRP [mg/L, M (Q1, Q3)]	11 (2.7, 27.5)	14 (0.7, 91)	0.841	0.401
WBC in pleural and ascitic fluid [×10^9^/L, M (Q1, Q3)]	1,377.5 (472.3, 2,788.3)	553 (186, 3,577)	0.25	0.803
Neutrophil ratio in pleural and ascitic fluid [%, M (Q1, Q3)]	53.5 (12.5, 82.5)	45 (27,84)	0.15	0.881
Total protein in pleural and ascitic fluid [g/L, M (Q1, Q3)]	27.1 (23.0, 30.0)	29.3 (23.2, 38.3)	0.575	0.565
Pleural and ascitic fluid test results consistent with clinical findings [case, %]	3 (50.0)	23 (85.2)	-	0.093^a^
add new anti-infective drugs	4 (100)	11 (47.8)	-	0.484^a^
reduce anti-infective drugs	0 (0)	6 (26.1)	-	0.16^a^
remain unchanged	0 (0)	6 (26.1)	-	0.16^a^
In-hospital mortality ^b^	2 (33.3, 2/6)	3 (15.0, 3/20)	-	0.216^a^

^a^Fisher’s test; ^b^Mortality comparison was calculated in line with the first time mNGS test in patient with multi-time mNGS tests.

### Clinical coincidence of mNGS tests in exudate and transudate groups

The positive rate of bacteria was observed great higher in the exudate group than in the transudate group (70.0% *vs*. 41.7%, *P* = 0.158) than those in the transudate group. At the same time, the mean specific reads number (130 *vs*. 3, *P* = 0.009) were also discovered significant higher in the exudate group. In addition, the coincidence between detected pathogens and clinical findings was also higher in the exudate group, but no statistically significant difference was observed (66.67% *vs*. 50.00%, *P* = 0.483) (**Table 3**).

**Table 3:** Comparison of some observation indicators of pleural and peritoneal effusion exudate and transudate.

Results	Exudate group (N=30 samples)	Transudate group (N=12 samples)	Z value	P value
Bacteria [sample, %]	21 (70.00)	5 (41.67)	–	0.158^c^
Virus [sample, %]	7 (23.33)	7 (58.33)	–	0.67^c^
Fungi [sample, %]	4 (13.33)	1 (8.33)	–	1.000^c^
Negative [sample, %]	5 (16.67)	4 (33.33)	–	0.406^c^
Mean specific sequence number [reads, M (Q1, Q3)]	130 (13, 4,047) ^a^	3 (1, 3.5)^b^	2.606	0.009
Consistence with clinical findings [sample, %]	20 (66.67)	6 (50.00)	–	0.483^c^

^a^refers to 35 pathogens; ^b^refers to 19 pathogens; ^c^refers to Fisher’s test.

## Discussion

Pleural or peritoneal effusions are common complicated symptoms. The causes of pleural and peritoneal effusions in children are complex and can be primary or secondary infections or a variety of non-infectious diseases such as liver diseases, heart diseases, and tumors (Hildreth et al., 2009; Giefer et al., 2011). Previous studies have found that approximately 14.9% of peritoneal effusions in hospitalized children were associated with infection (Karnsakul et al., 2017). Utine et al. reported that among 492 hospitalized children with pleural effusion, the rate of secondary infection with pleural effusion was up to 77.4% (Utine et al., 2009). But for a long time, clinicians have placed additional emphasis on the therapeutic value of effusion drainage, as well as its diagnostic value for tumors or immunological diseases. The significance of pleural or peritoneal effusions in etiological diagnosis has not been completely realized because of the poor positive rate of conventional pathogenic tests. Although mNGS offer considerable promise for the detection of potential pathogens in such biological samples, there haven’t been any relevant reports on the clinical significance of their etiological diagnostics.

Serum PCT level is related to the severity of infection (Bartoletti et al., 2018) and has a certain predictive effect on thoracoabdominal infection (Lin et al., 2009; Su et al., 2013). In this study, the median serum PCT level was as high as 16.0 μg/L, and 34.3% patients had septic shock, which indicated that the possibility of severe infection or pleural and peritoneal effusion superimposed with infection was high in this group of children. Tumor is a common underlying disease of sepsis in PICU. Due to the weak immune barrier function, it is easy to lead to multi-site or multi-pathogen infection and poor prognosis. In this study, about 81.25% of the children had underlying diseases, of which tumors accounted for 50.00%. Therefore, identifying the pathogen as soon as possible is the key to improve the prognosis.

To improve the pathogen positive rate and pathogen diagnosis accuracy, clinicians can collect samples from multiple suspected infected sites for mNGS pathogen screening (Chien et al., 2022). As one of the most common sample types, blood has the easiest access for mNGS testing and is the most important sample type for joint analysis in this study. It was reported that the positive rate of mNGS test of blood samples in critically ill adult patients hospitalized in ICU was 5~6 times that of conventional methods (41.3% *vs*. 7.9%), and co-infection could be identified (Geng et al., 2021). Similarly, blood mNGS tests in children with suspected sepsis in PICUs can detect a variety of pathogens (Yan et al., 2021). In this study, the positive rate of mNGS test in blood samples was nearly eight times that of traditional tests (83.33% *vs*. 11.11%), and the proportions of 1-3 and 4-6 pathogens detected were 72.22% and 11.11%, respectively. It was reported that the positive rate of mNGS test in blood samples of adult patients with sepsis was 62.0% (Chien et al., 2022), while a higher positive rate (83.33%) of children with sepsis was observed in this study. However, no mNGS related studies of pleural or peritoneal effusions have been reported so far. This study showed that the positive rate of mNGS test in pleural effusions and ascites from children with sepsis was 78.8%, which was great higher than it (14.3%) in traditional culture. Furthermore, multi-type pathogens were detected by a single test in 42.86% (18/42) samples. Previous studies have shown that mNGS test has higher accuracy and specificity than the conventional methods (Gu et al., 2021), and this study showed a 66.7% agreement rate between the two methods in the regular pathogenic tested positive samples. It can be concluded that mNGS test is much more sensitive than conventional tests and can provide more comprehensive results in the pathogenic detection of pleural effusion and ascites samples. However, due to the technical limitations such as influence cause by background microorganisms in sampling and experimental progress, higher positive rates are not always better. The detection of pathogens in accordance with the clinician’s judgment is of the utmost concern. In this study, 78.79% of mNGS tests returned pathogens consistent with clinical judgments, a small number of potential false positives due to technical limitations were acceptable.

Generally, the same pathogen detected in samples from multiple sites or multiple samples from the same site is considered more likely to be the pathogen responsible for the infection. In this study, we discovered that the proportion of the same pathogen detected in pleural effusion or ascites and blood was relatively high (69.23%), which not only indicated that the infectious foci in pleural effusions or ascites and blood may be homologous, but also suggested that the results of blood mNGS detection had a good reference value for judging whether pleural and peritoneal effusion was secondary infection in children with sepsis. Judging from the types of pathogens detected, G- bacteria are predominant, with *Klebsiella pneumoniae* being the most common. *Klebsiella pneumoniae* is a normal colonized bacteria that present in human gut and respiratory tract. Factors such as infection, intestinal microecological imbalance, and immune barrier disruption can cause conditional pathogenesis of *Klebsiella pneumoniae*. Previous studies have shown that *Klebsiella pneumoniae*-related sepsis can significantly increase the risk of systemic multi-site infections (Li et al., 2021), and thoracoabdominal membranes are the potentially affected tissues (Ren et al., 2020; Ameer et al., 2022). Interestingly, *Klebsiella pneumoniae* was only detected in multiple samples at the same time. However, some pathogens detected in pleural effusions or ascites samples by mNGS were completely different from those in any other samples. For example, eighteen pathogenic organisms were only positive in pleural effusions or ascites, inferring that they might originate from adjacent foci of infection such as the lungs or digestive tract. Besides, 15.4% and 16.7% of the blood and deep sputum samples were negative, but the corresponding pleural effusions or ascites were positive. In spite of only common pathogens were detected in this study, some rare and diagnostic pathogens could also be detected in pleural effusions or ascites (Gupta et al., 2018). Thus, the results of mNGS should be carefully analyzed in combination with clinical conditions. In addition, mNGS is highly sensitive to both cytomegalovirus and Epstein-Barr virus (Duan et al., 2021), and this study also showed certain positive rates for pleural effusions or ascites.

To further investigate the clinical significance of the mNGS results in pleural effusions and ascites, the clinical characteristics and pathogen composition of each group were analyzed in multiple groups and compared in this study. First, samples were divided into pathogen-consistent and pathogen-inconsistent groups according to whether the detected pathogens in pleural effusions or ascites are consistent with the test results of other sample types (**Table 2**). PCT is a common indicator to determine bacterial infection, and several studies have shown that PCT levels are correlated with the abundance of pathogens detected in the blood of children with suspected sepsis in the PICU (Yan et al., 2021). In this study, the median PCT in the pathogen-consistent group was great higher than that in the pathogen-inconsistent group, which may be related to the relatively high proportion of pathogenic bacteria detected. Furthermore, it’s worth noting that the degree of clinical coincidence in pathogen-consistent group is better than it in the pathogen-inconsistent group (85.2% *vs* 50%, *P* = 0.093) as well as the mortality (15.0% *vs*. 33.3%, *P* = 0.216). This suggests that a combined multiple-sample test may have possible advantages in improving clinical diagnosis rates and prognosis, and the consistent pathogens proved by multiple-sample should be placed high priority in clinical practice. Certainly, further studies with larger sample size are needed to verified this view.

In order to determine whether the original character of pleural effusions and ascites could affect the pathogen detection of mNGS test, the pleural effusions and ascites samples were divided into exudate and transudate groups. Although fluid exudations are mostly caused by inflammation, they can also be caused by various other reasons, such as malignant tumors, connective tissue diseases, and pulmonary embolism (Burgess, 2004). Thus, their suggestive effects on infection are limited. In this study, the exudate group had a higher positive rate of bacterial infections than the transudate group, suggesting that bacterial infection is still the primary cause of pleural or peritoneal fluid exudation in this group of children. In addition, the clinical coincidences of these two groups were similar (73.3% and 67.7%), indicating that mNGS results have good clinical value regardless of exudations or transudations.

## Conclusions

In this study, we analyzed mNGS tests of pleural effusions and ascites from 32 cases of children with sepsis or severe sepsis, and a high positive rate of mNGS tests was observed in pleural effusions and ascites. mNGS can improve diagnostic accuracy and particular attention should be paid to interpreting the clinical significance of pathogenic organisms with high specific sequence numbers. mNGS test using contemporaneous samples from multiple sites can help improve clinical diagnostics, and pathogens consistent in different sample types have important clinical significance. However, the identification of exudation or transudation alone is of limited value in determining thoracic and abdominal infections. Since this study is retrospective and the sample size is small, potential case selection bias may have some impact on the findings, and further sample size augmentation is required in a future study.

## Data availability statement

The datasets presented in this study can be found in National Genomics Data Center (NGDC) (https://ngdc.cncb.ac.cn/) with the project number of PRJCA013799.

## Ethics statement

The studies involving human participants were reviewed and approved by the Clinical Research Ethics Committee of Beijing Children’s Hospital with approval number of [2022]-E-019-Y. Written informed consent to participate in this study was provided by the participants’ legal guardian/next of kin. Written informed consent was obtained from the individual(s), and minor(s)’ legal guardian/next of kin, for the publication of any potentially identifiable images or data included in this article.

## Author contributions

SQ, GL, and HX contributed to the study design. GL, LW, XL and YZ drafted the manuscript. YW and HG contributed to sample collection. GL, XL and HL contributed to data collection and analysis. SQ and HX revised the manuscript. All authors contributed to the article and approved the submitted version. All authors read and approved the final manuscript.
